# ‘I'm a sick person, not a bad person’: patient experiences of treatments for alcohol use disorders

**DOI:** 10.1111/hex.12379

**Published:** 2015-06-25

**Authors:** Stacey L McCallum, Antonina A Mikocka‐Walus, Matthew D Gaughwin, Jane M Andrews, Deborah A Turnbull

**Affiliations:** ^1^ School of PsychologyFaculty of Health SciencesUniversity of AdelaideAdelaideSAAustralia; ^2^ Department of Health SciencesUniversity of YorkYorkUK; ^3^ Drug and Alcohol Clinical Liaison Service & School of Public HealthUniversity of Adelaide at the Royal Adelaide HospitalAdelaideSAAustralia; ^4^ Department of Gastroenterology and Hepatology & School of MedicineUniversity of Adelaide at the Royal Adelaide HospitalAdelaideSAAustralia

**Keywords:** alcohol use disorder, continuity of care, patient satisfaction, qualitative research, treatment need

## Abstract

**Background:**

Emerging research indicates that standard treatments for alcohol use disorders may not fully meet the needs of patients with co‐occurring severe mental health symptoms. Investigating health quality indicators may provide insight into how current treatment might be improved.

**Objective:**

To better understand the experiences of patients receiving treatment for alcohol use disorders and compare the experiences of patients with and without co‐occurring severe mental health symptoms.

**Design:**

Cross‐sectional qualitative research design using semi‐structured interviews methods and framework analysis approach.

**Setting:**

Inpatient hospital, outpatient service, inpatient detoxification clinic and a residential/ therapeutic community.

**Participant's:**

Thirty‐four patients receiving treatment for an alcohol use disorder.

**Main variables studied:**

Themes relating to patients' experiences of continuity of care, treatment need and satisfaction with treatment were studied. The qualitative data were divided into two groups: patients with (*n *=* *15) and without (*n *=* *19) severe mental health symptoms.

**Results:**

Five themes relating to patient satisfaction with treatment were identified, including: perceived effectiveness of treatment, supportive relationships, specialized but holistic care, patient autonomy and continuity of care. A diverse range of patient treatment needs, staff and service continuity and stigma were also identified as major themes. Five basic themes were identified as more critical to the experiences of patients with severe mental health symptoms.

**Discussion and conclusions:**

Findings suggest that patients look for supportive relationships with others, to be involved in treatment decisions, effective specialized and holistic approaches to care and a non‐judgemental treatment environment.

## Introduction

Alcohol use disorders (AUDs) are one of the leading causes of disability and death in Australia, affecting up to one in 15 persons.^1^, ^2^ AUDs are a chronic relapsing condition and patient prognosis is poor.^3^ Many patients do not respond to treatment and 50–80% dropout of treatment before the recommended treatment duration.^4^ Accordingly, AUDs place heavy recurrent burden on the health‐care system.^5^ In Australia, AUD treatment episodes have risen from 42 000 in 2001–2002 to more than 67 000 in 2009.^6^ A particularly vulnerable group of patients in AUD treatment are those with additional severe mental health symptoms (SMHS). Approximately 50% of patients in AUD treatment also present with SMHS, the most common being depression and anxiety.^7^ These patients are found to be susceptible to poor treatment prognosis, relapse to alcohol, treatment readmission and poorer quality of life when compared to patients with only an AUD.^8^, ^9^, ^10^

Health‐care strategies emphasizes the need to enhance the patient experience of AUD treatment, to improve treatment course and patient outcomes.^11^, ^12^, ^13^ Strategies highlight the need to better understand the patient perspective of existing service delivery models, specifically in areas of continuity of care (CoC), treatment need and patient satisfaction.^14^ These are recognized key health‐care objectives and are considered indicators of treatment quality.^15^ In the context of AUD treatment, patient satisfaction has been linked to improved patient retention^16^ and improved treatment outcomes such as reduced drinking days and improved clinical status.^17^ Similarly, CoC is thought to improve patient AUD treatment course and outcomes^18^; however, there is a lack of quality evidence on this topic. Although some researchers have proposed that patients in AUD treatment with additional SMHS experience poorer CoC^19^, ^20^ and treatment satisfaction^21^ when compared to patients with an AUD alone, little research has been carried out.^22^, ^23^, ^24^

Continuity and appropriateness of AUD treatment has, until now, been largely assessed using administrative service‐use data sources. However, recent research advances indicate that administrative assessments lack practicality and do not fully capture the quality or patient's perspectives of existing treatments.^14^ There is also limited information on whether current services address the needs of patients in AUD treatment and how patients are being transferred within the treatment system.^14^ Nonetheless, guidelines now suggest that the patient perspective of care should be used to assess the quality of AUD treatment. The patient's perspective of care is considered most important when attempting to improve patient treatment outcomes.^25^

The aim of this study was to explore the patient experience of AUD treatment in areas of CoC, treatment need and patient satisfaction. This study also sought to compare experiences of patients with and without SMHS, to identify whether specific issues might relate to the complex needs of these patients. Traditionally, patients with co‐occurring problems are required to meet full diagnostic criteria. However, this study focused on symptoms of mental illness, rather than a diagnosis, as symptoms may nonetheless impact significantly on a patient's functioning and treatment outcomes.^26^ Furthermore, previous research suggests that patient treatment needs differ across various settings.^27^ Accordingly, this study recruited patients from four different service settings. To the authors' knowledge, this study is the first study to qualitatively investigate patient experiences of CoC, treatment needs and satisfaction in AUD treatment, comparing those with and without SMHS across a variation of treatment settings.

## Methods

### Participants

This study used a theoretical sampling strategy^28^ to recruit patients presenting to an inpatient hospital, outpatient service, inpatient detoxification clinic and a residential/therapeutic community. Patients were required to (a) meet DSM‐5 criteria of an AUD, (b) be engaged in AUD treatment, (c) be proficient in English and (d) have alcohol as the main substance of concern. Potential participants were excluded if they (a) had consumed alcohol <5 days prior to assessment, (b) were cognitively impaired or (c) were too physically or psychologically unwell, as assessed by treatment staff. The on‐going theoretical sampling process indicated that patient gender, previous treatment history and treatment setting affected patient treatment experiences. Sampling procedures therefore aimed to recruit an equal proportion of patients with each of these characteristics.^29^

### Procedure

A priori themes (patient perspectives of CoC, treatment need and patient satisfaction) were informed by key questions raised in the Australian National Comorbidity Initiative report.^14^ Academic literature and theoretical models of health‐care delivery were also reviewed to establish a basis for the themes.^30^, ^31^, ^32^ A framework analysis approach was considered most suitable to explore, understand and explain patient experiences of health care within the highly objectified aims.^33^ It is also considered a systematic, flexible and dynamic approach to analysis of qualitative health‐care data.^34^ Approval was received from the Royal Adelaide Hospital and the University of Adelaide Research Ethics Committees. Recruitment began at the tertiary hospital in October 2013 and ended in February 2014. Patients were recruited at the residential service in November 2013 and the withdrawal unit in February 2014. Staff were consulted to identify potential participants based on the inclusion and theoretical sampling criteria. Patients who provided voluntary informed consent became participants. Interviews were conducted in a private space at the service (*n *=* *30) or over the telephone (*n *=* *4).

After interviews, patients completed the Depression Anxiety Stress Scale (DASS‐21)^35^ to assess mental health symptoms. The DASS‐21 contains 21‐items for symptoms of depression, anxiety and stress experienced over the past week. The DASS‐21 has good construct validity and reliability and has been applied in Australian AUD treatment samples.^36^ Patients who scored in the ‘extremely severe’ range for depression and/or anxiety were grouped as having SMHS. This cut‐off is used when patient symptoms warrant clinical intervention and treatment.^35^ The investigator was unaware of the patient mental status at the time of interviews (except as disclosed incidentally during the interview) to reduce the researcher bias on the data.

### The interview

The interview was developed by the primary investigator and last author based on interview protocol recommendations.^37^, ^38^ These guidelines suggest the use of open‐ended questions, icebreakers, prompts, ordering of questions based on difficulty and flexibility to deviate from set questions.^37^ The interview was designed to be inductive and deductive to examine a priori themes and allow patient‐driven themes to arise.^39^ An example interview item was ‘*what parts of your treatment have been working well for you’?* The investigator, a provisional psychologist and doctoral student, trained in interviewing and reflective listening conducted all interviews. Most interviews averaged 25 min in length but ranged from 15 to 50 min).

### Data analysis

Data were considered saturated after 30 interviews; however, two further interviews were conducted at the outpatient and withdrawal service to test emerging data regarding level of patient treatment experience, within these specific services.^40^ Results confirmed the data that had emerged from interviews at the tertiary hospital.

**Figure 1:**
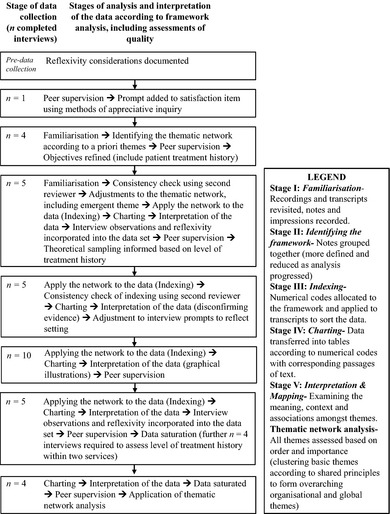
Framework analysis approach for the entire sample (*n* = 34).

The data were divided into two groups: patients with (*n *=* *15) SMHS and those without (*n *=* *19) and were analysed separately according to the framework method.^33^ Framework analysis involves five interconnected stages that occur throughout data collection, analysis and interpretation.^33^ Figure 1 illustrates the framework analysis method for the entire sample and provides details on each stage of analysis. Consistent developments to the framework and interview schedule were required to accommodate treatment settings and to test emerging data. For example, objectives initially aimed to focus on *current* treatment experiences; however, on‐going analysis found patients made judgements by drawing comparisons to previous treatments. Therefore, research objectives were refined to allow for inclusion of this data. Final interpretations of the data indicated a complex and interrelated series of *a priori* and emergent themes, and a thematic network analysis^41^ was also conducted.

Quality of the data was assessed to improve rigour and trustworthiness of findings.^42^ A second reviewer evaluated analysis at two separate time points, to assess the consistency of findings. The second reviewer independently coded 10 transcripts during the *familiarization* stage and allocated numerical codes to a further five transcripts during the *applying the analytical framework* stage to improve the consistency of interpretations.^43^ Reviewers assessed the level of agreement between independent codes and indices where one minor discrepancy was identified. The second reviewer reported problems in discerning whether data related to current or previous treatments and interior or exterior staff/services, which was particularly important in the analysis of CoC data. To resolve this issue, reviewers met frequently to clarify information. Qualitative rigour^43^ was monitored with an audit trail documented by the primary investigator and used during meetings with last author to discuss the development of themes and ideas. Quality was also assessed using methods of disconfirming evidence^44^, ^45^; a measure of validity where data contrary to major findings is investigated.^42^

## Results

**Figure 2:**
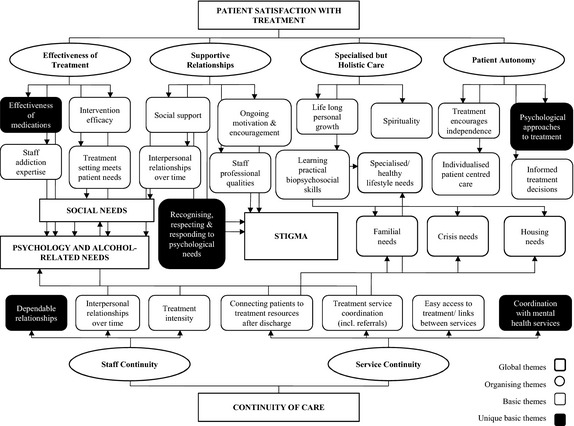
Thematic network illustrating qualitative data from the total sample (*n* = 34).

Table 1 includes the demographic and clinical characteristics of patients in the sample (*n *=* *34). Figure 2 illustrates the thematic network that was developed from framework analysis of the data, including the five unique basic themes that emerged from interviews with patients with SMHS.

**Table 1:** Demographic and clinical characteristics of patients in the total sample (*n *=* *34)

Variable	*n*/M	%/SD
Gender		
Male	22	65
Female	12	35
Age (years)	44.25	10.92
Treatment setting		
Inpatient hospitalization	10	29
Outpatient	7	21
Inpatient detoxification	7	21
Residential Therapeutic Community	10	29
Ethnicity		
White/caucasian	33	97
Other	1	3
Marital status		
Never married	17	49
Separated	7	21
Divorced	7	21
Married	3	9
Highest education level		
High school	20	59
Tertiary	14	41
Usual employment pattern		
Full‐time	7	21
Part‐time	3	9
Casual	5	15
Student	1	3
Retired/disability pension	9	26
Unemployed	9	26
Perceived AUD length (years)	12.38	7.85
Severe mental symptom severity (*n *=* *15)		
Depression^a^	14.73	3.58
Anxiety^a^	12.93	4.37
Stress^a^	15.53	3.34
Patients with single AUD (*n *=* *19)		
Depression^a^	6.12	4.04
Anxiety^a^	3.18	3.07
Stress^a^	6.0	4.27

a Depression Anxiety Stress Scale (DASS‐21), scale range [0–21].

### Treatment needs

Three core areas of need were identified: AUD related, psychological and social needs. Alcohol‐related needs were those related to cutting back or quitting alcohol. Patients described requiring assistance with the medical management of withdrawal and cravings, trialling medications, breaking the cycle of drinking through inpatient stay and being connected to abstinence‐based programs.

Patients also described needing assistance with psychological symptoms. Patients discussed their desire to learn about the role of mood, anxiety and stress on alcohol use and to develop other coping strategies and methods of emotional regulation. Patients also discussed the positive impact of settings that nurtured their sense of acceptance. Patients mentioned other needs that were significant at the individual level, but were less common across the total sample. These included the following: housing, crisis, familial and spiritual/healthy/lifestyle needs.

#### Recognizing, respecting and responding to psychological needs

Patients with SMHS expressed a need for psychological help. These patients indicated they needed medications to reduce mental symptoms, psychological diagnostic assessments and access to treatments for mental illness. A number of patients with SMHS described a dislike of medication‐focused treatment and a preference for psychosocial treatment options:

It's just that I get this very bad depression and everybody just wants to put me on a drug that kills it. You know they are just focusing on the alcohol and not the psychology. If they focused on the psychology then alcohol wouldn't be a problem. I need counselling. I need someone to talk to

[Male, 65, SMHS, Inpatient Hospitalisation].

### Patient satisfaction with treatment

#### Perceived effectiveness of treatment interventions

Patients looked for immediate improvements to their physical and mental health and expressed feeling frustrated when interventions lacked immediate efficacy. Patients also discussed the importance of treatments that offered advice, tools and resources that improved their motivation to cut down or quit alcohol. A dissatisfaction described by patients was interactions with staff^1^ who they felt had little understanding of addiction, which increased feelings of self‐blame. Patients often described the hurtful effects of staff who simply ‘told’ them to stop drinking, which undermined their self‐confidence to change their drinking habits:

‘I was seen by the head of [the general hospital department (not drug and alcohol service)] and he just berated the crap out of me for drinking and you know, not particularly helpful. That's not really going to make someone who's not feeling very good about themselves and their drinking habits stop drinking, just because someone slaps you around your head a little bit.

[Female, 37, single AUD, Inpatient Hospitalisation]

Patients with complex needs valued general inpatient services, as they ‘broke the cycle of drinking’. Some patients in non‐specialized settings (i.e. inpatients in hospital) described a desire to be transferred to a specialized service, explaining that they would feel more accepted. Similarly, some inpatients felt that their symptoms were often minimized by general staff in hospital and this related to feelings of being unwanted.

#### Effectiveness of medication for both psychological and alcohol‐related symptoms

Patients with SMHS frequently cited dissatisfaction with the management and efficacy of medications. These patients looked for relief from both psychological and alcohol‐related symptoms and described feeling uncomfortable when medications did not appear to reduce presenting symptoms:

‘Another really frustrating thing is that they're only giving me half of the Valium I need. They haven't provided me with enough medication during withdrawal. They should be trying to make this as comfortable for me as possible

[Male, 55, SMHS, Inpatient Hospitalisation]

#### Supportive relationships with others

A major theme discussed by patients was the importance of building supportive relationships. Patients expressed a desire to work with staff who possessed qualities such as empathy, understanding, trust, respect and expertise and described feeling accepted in these relationships. Patients who perceived staff to be non‐judgemental in their approach described that this reduced their feelings of shame. Patients were dissatisfied with members of staff who lacked the aforementioned qualities, which patients felt made them feel isolated, guilty and misunderstood:

‘Not to make assumptions. Um you know doctors [general medical doctor] make assumptions about things. That really aggravates me. They just see me as an alcoholic

[Male, 65, SMHS, Inpatient Hospitalisation]

Supportive relationships were enhanced when they continued over time. This was particularly important for outpatients who described that a strong bond with staff acted as a motivator to abstain from alcohol so as not to disappoint staff. Patients who described past experiences of social isolation discussed the value of supportive relationships with other inpatients in treatment. These relationships enabled patients to gain advice from each other's experiences and were instrumental in helping patients feel that they were not part of a stigmatized group:

‘For me, I've never had anyone to let all this out to, someone who was in a similar situation and I mean we are all kind of in the same boat here, like all taking drugs and alcohol… it's just that support from the whole community. You feel really welcome and you don't feel out of place. Everyone has their ups and downs but you offer them support and they will offer you support if they can. You can relate to people here. There is no judgement you know

[Male, 45, Single AUD, Residential Therapeutic Community]

#### Specialized but holistic care

All patients in the sample expressed satisfaction when they felt that their treatment centred on their addiction while also taking a holistic approach to meeting their other complex needs. Patients emphasized the importance of learning a variety of skills to assist them in a number areas of functioning. Patients who had previously sought treatment believed that to recover from addiction were life‐long work and expressed a desire for tools that encouraged their on‐going personal growth. Patients also discussed the importance of spirituality by engaging in behaviours that connected oneself to an inner goodness.

#### Patient autonomy

Patients valued being involved in decisions about their treatment. Patient autonomy emerged as a theme for the majority of patients across all settings. This approach enabled patients to direct treatment in a way that encouraged their independence and satisfied their individual treatment needs. Some patients reported being dissatisfied when they felt uninvolved in the treatment process, which increased their feelings of inadequacy and increased perceptions of judgement:

‘Regardless of whether I was in the state of mind for it or not, this involves me and what I'm doing and I need to know what is going on. You know we're not stupid because we're addicts and alcoholics, don't play us like dummies

[Female, 39, SMHS, Residential Therapeutic Community]

#### Psychological approaches to treatment

It was important for patients with SMHS that staff respected their desire for psychological approaches to treatment. These patients often believed that if their mental health symptoms improved they would be able to reduce or quit alcohol consumption. Patients expressed a desire for information on how to access psychological treatments and what these would entail.

### Continuity of care (CoC)

Analysis revealed that CoC was closely associated with patient needs and satisfaction, where good or poor CoC practices often influenced whether patients felt their needs had been met or if they were satisfied with treatment. Analysis indicated two organizing themes of CoC: staff and service continuity and within these five basic themes: treatment intensity, staff relationships over time, continuity after discharge, continuity across services (including communication) and access and transfer between services.

#### Staff continuity

Patients in hospital and at the outpatient service described the importance of contact with the same staff member over time. Patients said that staff who had a good understanding of their history ‘they know my story’ enabled more productive treatment sessions. Patients in all settings emphasized the importance of treatment intensity, which was described as whether staff had made substantial efforts to understand their complex treatment histories and needs.

#### A dependable relationship with staff over time

Patients with SMHS discussed the need for staff to be dependable and reliable. Issues relating to frequent changes of staff and appointment cancellations were often raised. Some patients described situations where a breakdown in the continuity of the relationship led to a relapse in alcohol use.

#### Service continuity

Difficulty accessing treatment led to patients being dissatisfied, particularly amongst those at the residential service and those from rural areas. Not only did patients express frustration with the length of waiting lists, but they also discussed that access requirements (i.e. regular contact with the service) were burdensome. Difficulty accessing treatment distressed some patients; it influenced one to attempt suicide and for others it led to drinking more alcohol:

‘I got told it was going to be a 3‐month wait and it got to 3 months and they said I was only half‐way on the list and I remember thinking I wasn't going to make it and I tried to commit suicide. It was just, it was awful. I knew this place was around 2–3 years ago but obviously I had a house, a mortgage and I was the main income earner. I knew I needed an intensive program… but it was like I had to wait until everything was gone before I could do it

[Male, 37, single AUD, Residential Therapeutic Community]

Coordination of different services/teams/clinicians emerged as a significant issue for patients in all settings. Patients said they needed their various providers to communicate regularly, follow through with referrals and work together to offer integrated treatment. Patients described that more coordination in treatment lessened their confusion and made them more willing to engage. Inpatients specifically described feeling as though treatment had not adequately prepared them for discharge. Most patients said that CoC practices were important for their long‐term recovery; however, some disagreed. These patients outlined a preference for various treatments to be segregated and explained that they were capable of coordinating each treatment themselves. Similarly, patients seeking treatment for the first time believed that being connected to other resources after discharge was not important. Such patients described a desire to focus on personal goals and aspirations to achieve abstinence.

#### Connection and coordination with mental services

Patients with SMHS emphasized the importance of coordination among services for addiction and mental illness. Patients frequently reported difficulties when trying to access psychiatric treatment whilst in substance treatment settings, which often led to neglecting their mental health symptom needs.

‘What I have found difficult is that I have post‐traumatic stress disorder and so when I came in here I had a very structured plan of what I needed to do to get well. I have found myself a private psychologist but I have needed to be in a safe environment and be involved in this program. I have been restricted in being able to see my psychologist and they haven't been flexible. I have needed them to work together and they haven't

[Female, 39, SMHS, Residential Therapeutic Community]

### Stigma

Analysis of all data sources highlighted the negative impact of stigma that emerged as a global theme. Patients described how stigma affected the process of seeking treatment and expressed a desire for more public awareness in the wider community.

I want people to know that I'm not just a homeless bum on the street who is an alcoholic. I mean I'm 32 and I'm just a standard young female who suffers from chronic alcoholism. I am a sick person, not a bad person. A lot of people don't seek treatment because they don't want to admit they're an alcoholic or an addict because of the stigma and a lot die young because of accidents or they kill themselves because of the stigma. If they had just realised they were sick like everyone else.

[Female, 32, single AUD, Residential Therapeutic Community]

Patients described ways in which approaches to care worsened or alleviated their pre‐existing feelings of indifference. Stigma was described as greatly affecting the quality of relationships formed during treatment. Patients believed that receiving treatment in group settings reduced their perceptions of being different. However, some patients felt stigmatized and judged by staff. Patients described this stigmatization as not necessarily overt, but rather a ‘vibe’ or mere sense of being looked down upon. Notably, it was observed that regardless of whether stigmatization was intended, these feelings were very powerful for patients and often determined whether they wished to continue treatment.

## Discussion

The primary aim of this study was to gain better insight into patient views of AUD treatment, in relation to CoC, treatment needs and patient satisfaction. The strengths of this study lie in its rigourous qualitative design, systematic method of analysis, diverse sample of *n *=* *34 patients and objectified aims to address issues in AUD treatment. To the authors’ knowledge, this is the first study to qualitatively explore CoC, treatment needs and treatment satisfaction from the patients’ perspective in AUD treatment, using methods of cross‐sectional analysis.

The findings highlight the importance to patients of strong and effective relationships with staff. The influence of supportive relationships is well recognized in the literature, where previous quantitative studies have linked staff–patient alliance to improved patient treatment course and outcomes.^46^ This study identified a notable link between the quality of relationships formed in treatment and patients’ perception of stigmatization. This finding agrees with previous quantitative research demonstrating that an increase in patient self‐stigma reduces patients’ willingness to seek treatment for substance abuse.^47^ Similarly, findings support research that some health‐care professionals are perceived by patients as lacking understanding and empathy and of being judgemental.^48^ The results indicate that patients value autonomy and patient‐centred approaches to treatment. This finding is in accordance with previous quantitative research demonstrating the positive impact of provider training in patient‐centred care (i.e. motivational interviewing) on the provider–patient relationship in primary care settings.^49^ A wide range of AUD treatment approaches exist, and it is well documented that certain treatments are beneficial for different types of patients.^3^ The data from the study reflected this, as patients were satisfied when they believed treatment was beneficial and effective at addressing their specific needs. This study identified that patients looked for treatments that were specialized in addressing addiction but also aimed treat a range of other needs. Areas of patient need commonly seen in AUD treatment settings include the following: medical, psychological, alcohol, social/familial, legal, drug and employment.^50^ Results from this study support the range of needs patients have in treatment, as patients identified that they required most assistance with AUD, psychological problems and social problems. The patients’ perspective of CoC in AUD treatment is currently not well understood. However, the literature in the area of CoC has described studies of consistent contact with staff, length of stay in treatment and access to services.^51^, ^52^ This study identified that patient's value consistent and intense contact with supportive providers over time, easy access to services, coordination and consistency amongst various providers/services and being connected to on‐going treatment resources after discharge.

Findings from this study also identified experiences that were more common to patients with SMHS in AUD treatment. The results indicate that patients with SMHS were frustrated when they perceived that medications are mismanaged or lacked efficacy. Previous studies also report such experiences, describing that patients with co‐existing and substance abuse and mental disorders are less compliant with medications than patients with just a single diagnosis.^53^ Similarly, there is minimal evidence to support the effectiveness of medication interventions for patients with co‐occurring diagnoses, as comorbidity is often an exclusion criteria in research trials.^54^ Results also suggested that these patients needed staff to value psychological approaches to treatment and required strong coordination between AUD and mental treatment services. This supports the growing recognition in the literature on comorbidity of the problems caused by separating services for addiction from services for mental illness. The finding also supports the negative impact that the separation of services has on patients’ capacity to access and engage with the treatments they require.^55^

The findings highlight the impact of past treatment experience on patient appraisals of treatment, particularly CoC. It was observed that patients receiving treatment for the first time displayed limited knowledge of AUD treatment services, appeared less motivated for change, were more inclined to reduce alcohol intake than aim for abstinence and were less interested in communicating their admission to their other health‐care clinicians or receiving further treatment after discharge, when compared to patients with previous treatment admissions. This finding supports previous studies, which have linked patient treatment readiness and addiction severity to patient satisfaction with treatment and outcomes.^56^, ^57^, ^58^, ^59^ Future quantitative research would benefit by investigating the impact of these variables on CoC in this treatment context. In addition, patients receiving treatment for the first time discussed non‐treatment related goals such as becoming a better parent or getting a job. This finding may point to the usefulness of social work services, life‐coaching or existential counselling as a suitable treatment approach for patients with less severe addictions and/or minimal motivation to change.

Findings, which illustrate the patients’ perspective of care, are worth considering by clinicians, researchers and policymakers aiming to improve patient experiences of AUD treatment. It is important to acknowledge that results from this study only reflect one perspective of care and improving treatment quality requires a mutual responsibility from providers and patients. However, the following suggestions may be helpful for providers in refining their clinical practice to enhance patient treatment experiences. In circumstances where AUD services do not permit the treatment of non‐alcohol‐related needs, providers are encouraged to network with services that do so. Providers are then encouraged to practice active referral and assertive follow‐up through organizing appointments, exchanging information, jointly developing treatment plans and maintaining regular contact to monitor patient progress.^60^ It is suggested that staff feel confident to identify, treat and respect patients’ mental symptoms for patients with SMHS. Providers are suggested to feel confident in offering treatment options and managing patient expectations through information on treatment access, suitability and effectiveness, particularly in regards to medications for patients with SMHS. A common dissatisfaction for SMHS patients in acute care was the need for immediate symptom relief from medication. Direct discussions of the purpose for medications, their side‐effects and likely effectiveness for both symptoms is likely to benefit patients’ expectations. Providers are also encouraged to equip patients with the information necessary to make decisions about their current and on‐going treatments and respect their preferred treatment approach. Patient‐centred‐care practices are crucial; however, providers must be cautious not to leave patients to navigate their way through services. Providers are encouraged to acknowledge on‐going and intense relationships with patients. Accordingly, to improve staffs’ capacity to form meaningful relationships with patients, it is suggested that all levels of staff have sufficient addiction training. From an organizational perspective, services might consider reviewing how staff are rostered and resourced, as a means to improve the consistency of staff for patients with SMHS. Addressing issues of high staff‐turn over and increasing patient contact with staff is likely to have a positive impact on patients with SMHS experiences of AUD treatment.

Despite such positive contributions, this study contained limitations. Patients were required to be abstinent from alcohol for at least 5 days to minimize the impact of alcohol withdrawal on mental health symptoms. This criterion therefore excluded patients who had prematurely discharged themselves or did not attend their appointment. Patients also were required to be engaged in treatment, thereby excluding those who were unable or did not wish to access treatment. Thus, it is possible that sample bias may have underestimated issues in accessing services or dissatisfaction at the commencement of treatment. However, sample bias may have been reduced as patients discussed their retrospective treatment experiences. Future research would benefit from recruiting patients on waiting lists or those who had left treatment early to determine whether significant issues were missed or underestimated. To enhance the utility of a second reviewer in qualitative data analysis, future studies should seek to appoint a reviewer who has sound knowledge of the specific clinicians, services and system under investigation in the study.

This study provides a framework for methods to improve patient experiences of AUD treatment in relation to CoC, treatment needs and satisfaction. Patients look for supportive relationships, to be involved in treatment decisions, effective specialized and holistic approaches to care and a non‐judgemental treatment environment. Patients require easy treatment access, intense contact with staff and coordinated treatment approaches. Although these findings do not represent the views of all patients in AUD treatment, findings give insight into the ways treatment providers, service managers and policy makers might enhance the patient experience of AUD treatment to improve patient treatment prognosis and outcomes.

## Sources of funding

The primary investigator, SLM completed all research data collection. Researcher time was supported by the University of Adelaide, Adelaide, South Australia, Australia.

## Conflicts of interest

No conflict of interests has been declared.

## Note

1 ‘*Staff’* is used interchangeably throughout the reporting of qualitative data and might refer to a patients’ treating doctor, psychologist, psychiatrist, nurse, counsellor, treatment receptionist or manager, etc.
